# Meat Quality Traits Using Gelatin–Green Tea Extract Hybrid Electrospun Nanofiber Active Packaging

**DOI:** 10.3390/foods14101734

**Published:** 2025-05-13

**Authors:** A. M. M. Nurul Alam, Young-Hwa Hwang, Abdul Samad, Seon-Tea Joo

**Affiliations:** 1 Division of Applied Life Science (BK21 Four), Gyeongsang National University, Jinju 52852, Republic of Korea; alam6059@yahoo.com (A.M.M.N.A.); buzdarabdulsamad@gmail.com (A.S.); 2 Institute of Agriculture & Life Science, Gyeongsang National University, Jinju 52852, Republic of Korea; philoria@gnu.ac.kr

**Keywords:** active packaging, electrospinning, hybrid nanofiber, physicochemical properties, antibacterial efficacy, taste traits

## Abstract

The adverse effects of polyethylene packaging waste on environmental pollution have driven academia to explore biodegradable active packaging (AP) solutions. In the present study, hybrid electrospun nanofiber (HENF) AP was produced using 30% gelatin (GE) combined with 1%, 2%, and 3% green tea extract powder (GTEP), termed HGGTNF. HENF was applied to Hanwoo beef as an AP to assess physicochemical, textural, microbiological, and sensory qualities in comparison to traditional polyethylene packing (PEP). The findings illustrate that the HGGTNF group maintained a significantly (*p* < 0.05) stable pH (5.71 ± 0.02–5.78 ± 0.01), lower drip loss (DL) (1.15% ± 0.00 to 1.20 ± 0.02%), and cooking loss (CL) (18.13 ± 0.03% to 19.91 ± 0.01%) compared to PEP (pH = 5.66 ± 0.02, DL = 1.21 ± 0.01%, CL = 20.26 ± 0.03%). Moreover, HGGTNF improved oxidative stability, especially at elevated doses (2% and 3%). In HGGTNF groups, there was a decreasing (*p* < 0.05) trend in thiobarbituric acid reactive substances (TBARS) (0.23 ± 0.01 to 0.26 ± 0.01 mg-MDA/kg), compared to the PEP group (0.29 ± 0.01 mg-MDA/kg). Oxidative stability improved the fatty acid profile, preserved color intensity (Chroma), and inhibited discoloration (*h°*) in HGGTNF (2% & 3%) compared to PEP. Furthermore, HGGTNF groups had stable meat tenderness and better chewiness than PEP. Stabilization of tenderness was due to diminished cathepsin activity (5822.80 ± 20.16 and 6009.80 ± 3.90 U/mg protein in the HGGTNF 2% and 3% groups, respectively). The HGGTNF 3% sample exhibited a decrease in total coliform counts (TCC) (0.74 ± 0.04 log CFU/g), total viable counts (TVC) (1.38 ± 0.05 log CFU/g), and total yeast and mold count (TYMC) (1.59 ± 0.06 log CFU/g) compared to other groups, indicating efficient antimicrobial efficacy. An increasing (*p* < 0.05) trend was observed in umami and richness taste traits for the HGGTNF 3% treated sample. The above findings underscore the potential applicability of HGGTNF as AP to enhance beef shelf life and meat quality attributes.

## 1. Introduction

The elevated protein, fat, and water content of beef renders it vulnerable to pathogen proliferation. Hence, the packaging is critical to keep the quality intact until it reaches consumers. Furthermore, consumer apprehensions regarding livestock meat are steadily rising owing to awareness of oxidation, spoilage, residual effects of packaging materials, and potential foodborne infections. To address these issues, scientists have attempted to incorporate natural additives into meat packaging materials to improve consumers’ health and reduce foodborne illness. In present practice, meat and meat products are predominantly packaged with polyethylene (PE), polypropylene (PP), polyethylene terephthalate (PET), etc. [1]. Casing, a form of traditional packaging composed of natural materials such as animal byproducts, collagen, cellulose, alginate, etc., is utilized for frankfurters [2]. Unceasing investigations are ongoing on sustainable and biodegradable packaging solutions, including nanofibers (NF) [3,4]. Biodegradable packaging materials are gaining prominence as sustainable alternatives to petroleum-based polymers, which generate a significant volume of plastic waste [5].

Electrospinning (ES) is an efficient technique for encapsulating bioactive materials, as it operates at ambient temperature (21–25 °C). The ES technique is used for fabricating nanofibers (NF), which are thin fibers ranging from 10 nm to several µm, and exhibit superior mechanical properties such as large surface area, porous and fibrous structure, flexibility, and encapsulation efficiency of different bioactive materials [6,7,8,9,10]. NFs are currently utilized both as protective films/mats and as filters to enhance the protection and mechanical and visual characteristics of food packing [8,11]. Gelatin (GE) NFs are widely used in the fabrication of composite materials, as individual polymers lack the requisite functional characteristics for sophisticated uses [12]. GE is an efficient biomaterial used for NF fabrication due to its superior biocompatibility, availability, low cost, strong aggregative nature, and, most importantly, it has been successfully used in previous experiments for fabricating NF for AP applications [6,13,14,15,16]. GE NFs have been investigated for their capacity to create antimicrobial active packaging (AP) films/mats while sustainably releasing encapsulated essential oils via their porous structure [17]. A significant trend in antioxidant and antimicrobial AP for meat is the decreased reliance on synthetic additives supplemented by natural bioactive materials. The predominant natural compounds are essential oils and plant extracts like rosemary, oregano, and tea [18]. The growing interest in edible films has been driven by consumer demand for safe, convenient, and stable food products [19].

*Camellia sinensis*, commonly known as tea, is a widely consumed drink globally [20]. Green tea extract (GTE) comprises several chemicals, including caffeine, theanine, theaflavins, theobromine, theophylline, and phenolic acids, with flavonoids, or catechins, being the most prevalent. GTE possesses well-documented antioxidant and antibacterial properties [21]. Research has been conducted by blending GTE directly into various meat and meat products to examine their microbiological and biochemical properties [22]. One study indicated that GTE addition shielded sausages against oxidative alterations [23]. A separate investigation indicated that sausages fortified with GTE exhibited superior storage and quality attributes relative to non-fortified groups [24]. ES was utilized to fabricate polyvinylpyrrolidone (PVP)/GTE composite NF mats [21]. PVP/GTE NF was successfully formed with uniformity and explained the ability of GTE incorporation in the ES NF. GTE has been recently applied in polyvinyl alcohol (PVA)-based NF to preserve fruits and showed superior antibacterial efficacy against *Staphylococcus aureus*, *Salmonella* spp., and total coliform count (TCC) [25].

The existing literature mainly focuses on the mechanical properties of AP. Furthermore, in most of the studies, more emphasis was given to oxidative stability and microbial parameters after application to the meat surface. There is a need to conduct research to analyze the physicochemical details and textural parameters of meat after the application of HENF AP. Moreover, it is important to analyze muscle degradation and microbial parameters as these are important for maintaining texture and the quality of meat. Previous studies have conducted sensory analysis after the use of AP on meat, but it is necessary to analyze important taste traits like umami and richness as well. To supplement previous research, we have fabricated GE/GTEP hybrid NF (HGGTNF) with 1%, 2%, and 3% concentrations of GTEP using the ES process. These HGGTNF APs were applied to Hanwoo Longissimus lumborum muscle (LLM) to compare the difference with traditional polyethylene packing (PEP) during refrigerated storage for 4 days at 2 °C. Physicochemical, nutritional, fatty acid profile, textural quality, oxidative stability, muscle degradation, microbiological parameters, and taste traits were analyzed on the samples using an electronic taste sensing system (TST) after the storage period. The hypothesis of this study was that HGGTNF AP, with increasing concentrations of GTEP, can stabilize the overall quality parameters of beef in comparison to PEP during refrigerated storage.

## 2. Materials and Methods

### 2.1. Materials

Gelatin (GE) powder (~225 g Bloom), acetic acid (99.5%), and citric acid (99%) were purchased from Sigma Aldrich, Seul, Republic of Korea. Green tea extract powder (GTEP) with 98% purity was purchased from Ahui Redstar Pharmaceutical Corp. Ltd., Ahui, China. Hanwoo Longissimus lumborum muscle meat was purchased from a local hypermarket in Jinju, South Korea.

### 2.2. Hybrid Nanofiber Fabrication

#### 2.2.1. Outer Layer

The HENF consisted of three layers: two outer layers (top and bottom part) made of gelatin, and an inner layer composed of gelatin with GTEP. The solution of the outer layer was prepared by mixing and dissolving 30% (*w*/*v*) gelatin in 40% (*v*/*v*) acetic acid at 45 °C/750 rpm for 12 h. The final mixture was later transferred into a 10 mL injection syringe and inserted into the Nano NC ES system (Geumcheon-gu, Seoul, Republic of Korea). The optimal ES parameters for fabricating smooth NF without beads were determined by trial-and-error runs and set up to a flow rate of 0.6 mL/h, a voltage of 21 kV, an emitter to fiber collector distance of 12.5 cm, a 24° angle, and a drum rotation speed of 600 rpm.

#### 2.2.2. Inner Layer

The solution of the inner layer was prepared by mixing and dissolving 30% (*w*/*v*) gelatin and GTEP in three concentrations (*w*/*v*) (1%, 2%, 3%). At first, GTEP was added to ethanol to prepare the stock solutions. Individual stock solutions were mixed on a magnetic stirrer at 1500 rpm/25 °C for 12 h to dissolve completely. Three treatments were made by mixing 10 mL of 1%, 2%, and 3% GTEP stock solutions with 20 mL of GE solution (2.2.1) and stirring at 1500 rpm/25 °C overnight until the solution became fully homogenized. The NF of the inner layer was overlapped on the previously made GE layer (2.2.1) with similar spinning parameters as the outer layer, except for a flow rate of 0.8 mL/h, 15 cm distance, and 18.7 kV voltage. Later on, another GE layer was overlapped onto the inner layer following the process defined in Section 2.2.1 for the outer GE layer.

#### 2.2.3. Taylor Cone, Bead, and Nanofiber Fabrication

During the fabrication of HGGTNF, trial-and-error runs were conducted to generate an effective Taylor cone. Moreover, choosing the correct needle size, referred to as gauge (G), was a critical factor, along with the distance from the needle tip to the drum collector, nozzle angle (°), drum rotation speed, and voltage supply (kV), to achieve bead-free NF for further analysis. All these data were documented in Supplementary Table S1 to assist further research on HGGTNF in meat or food products.

#### 2.2.4. Stabilization and Crosslinking of Prepared Nanofibers

**Figure 1:**
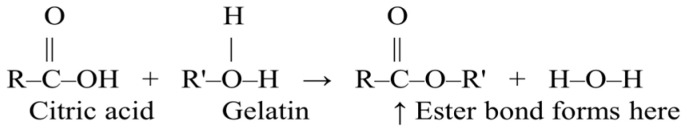
Chemical reaction involved in the crosslinking process.

For crosslinking (CRL), 1.25% (*w*/*v*) citric acid (CA) was used, with a modification of the methodology by Temel-Soylu [26]. The CA solution was prepared by mixing with deionized water and agitating at 750 rpm/25 °C for 12 h. The prepared NF mats were placed in the citric acid solution and kept in a hot air oven (Biofree, Bucheon-si, Republic of Korea) at 50 °C for 24 h. Afterward, HGGTNF samples were removed and placed into a sealed glass box with silica gel beads for 24 h to remove citric acid residues and allow drying. The chemical reaction involved in the crosslinking process is presented in Figure 1.

#### 2.2.5. Scanning Electron Microscopy Images

The morphology of the fabricated HGGTNF was analyzed using scanning electron microscopy (SEM) (FF-SEM, S-4800, Hitachi, Japan). HGGTENF samples were cut into small pieces (1 cm × 1 cm) and fixed onto a bronze stub using carbon tape, along with a thin layer of gold using a sputter coater [27]. HGGTNFs were observed under 2000× magnification to assess general morphology and bead detection.

#### 2.2.6. In Vitro Degradation

An in vitro degradation (IVD) was conducted in triplicate to evaluate the CRL of the HGGTNF in phosphate-buffered saline (PBS). HGGTNF samples (L 2 cm × W 2 cm) with initial weight (*W*_0_) were taken and then immersed in PBS (pH = 7.4). After 48 h of incubation at 37 °C, the samples were dried in a dry oven for 24 h and weighed again (*W*_1_). Equation (1) was used to calculate the percentage of degradation:

(1) $$IVD\;\%=\frac{W_{0}-W_{1}}{W_{0}}\times 100$$

#### 2.2.7. Swelling Parameters

A swelling test, conducted in triplicate, was used to assess the fluid absorption ability of HGGTNF. For this purpose, HGGTNF (L 2 cm × W 2 cm) was weighted (*W*_1_) and then suspended in PBS (pH = 7.4). After resting in PBS for 24 h at 37 °C, the samples were collected and blotted to dry out with cellulose filter paper. Subsequently, the blotted samples were weighted (*W*_2_), and swelling was determined with the following Equation (2):

(2) $$Swelling\;\%=\frac{W_{2}-W_{1}}{1}\times 100$$

#### 2.2.8. Mechanical Properties

A universal testing machine (TO-101 G, Testone Co. Ltd., Gyeonggi-do South Korea) was applied to measure the ultimate tensile strength (UTS) of HGGTNF. Samples (W 1 cm × L 4 cm) were subjected to tensile testing in triplicate at a stable rate of 6 mm/min, in accordance with ASTM D882.

#### 2.2.9. Application of Hybrid Nanofibers on Meat

LLM was sliced into 2 cm × 2 cm × 1 cm in width, length, and thickness, respectively, under aseptic conditions and wrapped using polyethylene (PEP), HGGTNF 1%, 2%, and 3% HENF, and stored in a covered Petri dish. All treated samples were stored at 2 °C in a refrigerator for 4 days before analysis. The period was set to 4 days, as fresh meat samples are kept in the retail display for a maximum of 3 days before being discarded.

#### 2.2.10. Nutrition Profile

The estimated components of moisture—dry matter (DM), crude protein (CP), and ash content—were analyzed using the techniques specified by the Association of Official Analytical Chemists [28]. Crude fat (CF) was determined using a modified method established by Folch, Lees [29].

#### 2.2.11. Fatty Acid Analysis

A gas chromatograph (GC) HP6890N equipped with an automatic sampler arm, HP7683 (Hewlett-Packard, Santa Clara, CA, USA), was employed to evaluate fatty acid analysis following the previous method of Alam et al. [30]. Following the extraction of CF (2.2.10), aluminum dishes (n = 3) were heated at 90 °C on a heat block, followed by the addition of 1 mL dichloromethane and gentle rotation (5 turns in the palm) for dilution. Then, 1 mL of the supernatant was transferred into a glass tube. Subsequently, 1 mL methanolic NaOH was added into the glass tube, and heated for 10 min. After cooling, 1 mL boron trifluoride (BF3) was added and again heated for 10 min. Following cooling, 3 mL hexane and 8 mL DIW were added and vortexed. Thesolution was then centrifuged at 1000× *g* (15 min, 4 °C), and a small quantity of Na_2_SO_4_ was added to dehydrate extra moisture for 1 min. Then, 2.5 mL of resultant fatty acid methyl ester (FAME) was separated into a glass tube, and 1.5 mL was transferred into a GC tube for fatty acid analysis. A 100-m SP2560 (Supelco, Santa Clara, CA, USA) equipped with a capillary column (0.25 mm internal diameter and 0.20 μm film thickness) was used to estimate the FAME extractions. FAME from the beef samples was refined using a temperature catalog with nitrogen serving as the carrier gas at a flow rate of 1 mL/min. The GC process involved a sequence of reactions conducted at specific temperature intervals: 50–180 °C with a heating rate of 10 °C/min, 180–220 °C at 5 °C/min, and 220–240 °C at 2 °C/min. The process was then maintained at a constant temperature of 240 °C for 20 min. The GC-equipped automatic sampler arm was utilized to perform eleven injections of 1 μL each. An estimation was made of the retention periods of fatty acid standards (Supelco 37 Components FAME Mix, Santa Clara, CA, USA). Ultimately, the FAME results were calculated as a percentage proportion of the overall fatty acids in the total peak area for corresponding fatty acids. The results were quantified as a percentage of the total fatty acid identified based on the total peak area. Atherogenic indexes were calculated by dividing the sum of saturated fatty acid (SFA) by the sum of unsaturated fatty acid (MUFA + PUFA), using the formula developed by Ulbricht and Southgate [31]: AI (atherogenic index) = [C12:0 + 4(C14:0) + C16:0]/[MUFA + PUFA].

### 2.3. Color Analysis

Colorimetric analysis was assessed in triplicate using a Konica Minolta CR-300 chromameter, made in Tokyo, Japan, according to King, Hunt [32]. Chroma (C*) and hue (*h*°) were determined with AMSA meat color standards and calculated using the following Equations (3) and (4) [32].

(3) $$C^{*}=\left(\sqrt{a^{2}+b^{2}}\right)$$

(4) $${h^{{}^{\circ}}=\tan}^{-1}\frac{b^{*}}{a^{*}}$$

### 2.4. Physicochemical Analysis

Meat pH was assessed (n − 3) with a digital pH analyzer (Orion Star^TM^ A211, Thermo Fisher Scientific, St. Bend, OR, USA).

Drip loss (DL) percentage was measured in triplicate using the method described by Joo [33]. Samples weighing approximately 25 ± 0.05 g were suspended using a metal clip in a PET box and refrigerated at 4 °C for 24 h. DL % was calculated using the following Equation (5):

(5) $$Drip\;Loss\left(\%\right)=\frac{\left(sample\;Fresh\;weight-Refrigerated\;weight\right)}{sample\;Fresh\;weight}\times 100$$

Cooking loss (CL) was assessed (n = 3) by calculating the percentage of weight reduction during cooking, following the methodology set by De Palo, Tateo [34]. Approximately 25 ± 0.05 g of meat was packed in a thin poly bag and cooked in water at 75 °C for 30 min. Chilled samples were weighed to determine CL% with the following Equation (6):

(6) $$CL\left(\%\right)=\frac{Precooked\;weight-Cooked\;weight}{Precooked\;weight}\times 100$$

Purge loss (PL%) was calculated (n = 3) by modifying the methodology described by Joo [33], and calculated using the following Equation (7):

(7) $$PL\left(\%\right)=\frac{\left(Damp\;filter\;paper\;and\;plastic\;film\;weight\right)-\left(Filter\;paper\;and\;plastic\;film\;weight\right)}{Meat\;sample\;weight}\times 100$$

Extract release value (ERV) was measured according to the method of Sadakuzzaman, Rahman [35], and the final volume was quantified and recorded in milliliters.

### 2.5. Oxidative Degradation Analysis

Peroxide value (POV) was measured following the methodology of Sadakuzzaman, Rahman [35]. Approximately 3 ± 0.05 g of sample was placed in a glass conical tube with a stopper. The flask was heated in a preheated water bath (60 °C, 3 min), followed by vigorous agitation for 3 min after adding 3:1 acetic acid:chloroform to dissolve the fat. The resultant liquid was filtered, and 0.5 mL saturated potassium iodide was added, followed by the addition of starch solution. Titration was then performed using sodium thiosulfate solution at a concentration of 25 g/L as the standard. Equation (8) was used to calculate POV in milliequivalents (meq) per kilogram of sample:

(8) $$POV\;\left(meq/kg\right)=\frac{S\times N}{W}\times 100$$

where S is the volume of titration (mL), N is the normality of standard, and W is the sample weight (g).

Thiobarbituric acid reactive substances (TBARS) were determined by mixing approximately 2 ± 0.05 g of minced meat with 18 mL of 1.2 M perchloric acid and 0.2 mL butylated hydroxy anisole (BHA) solution. The mixture was homogenized with a T25 digital Ultra-TURRAX homogenizer (Korea) for 1 min, then centrifuged at 1360× *g* for 10 min at 4 °C. The supernatant was filtered through cellulose filter paper. Subsequently, 2 mL of filtrate was added to 2 mL of 20 mM thiobarbituric acid (TBA) solution and left in a dark room for 16 h. Later, absorbance was measured at 531 nm using an Agilent 8453 UV-Vis spectrophotometer (Springvale Road, Mulgrave, VIC, Australia). TBARS values were expressed in mg MDA/kg.

### 2.6. Investigation of Textural Parameters

Following CL (Section 2.7), the same samples were used to determine Warner Bratzler shear force (WBSF) to quantify tenderness (kg/cm^2^), according to the protocol of Ismail, Hwang [36]. Approximately 1 cm of the sample was cut along the direction of muscle fibers with a load cell of 100 N (crosshead speed: 250 mm/min) using a Sun TP analyzer (Compact-100 II, Sun Scientific Co., Ltd., Tokyo, Japan).

Texture profile analysis (TPA) was conducted according to the protocol developed by Ismail, Hwang [36]. Meat samples (L2 × W2 × D1 cm) were packed in poly bags and boiled at 75 °C for 30 min in hot water. A flat pressure (No.1) was applied twice in an axial orientation using a rheometer at a crosshead speed of 60 nm/min and an ultimate strain of 60%, with a 10 kg load. The parameters assessed in TPA included hardness (N), springiness (cm), and cohesiveness. Gumminess (N) is defined as hardness × cohesiveness. Chewiness (N × cm) is calculated as hardness × cohesiveness × springiness.

### 2.7. Muscle Degradation Analysis

#### 2.7.1. Activity of Cathepsin B and L

Cathepsin B + L activities were assessed following the methodology of Li, Luan [37]. Approximately 1 ± 0.05 g of sample was blended with 5 mL of buffer (50 mM sodium citrate, 1 mM EDTA, and 0.2% Triton X-100 at pH 5.0) using a T25 digital Ultra-TURRAX homogenizer (Seul, Korea) for three intervals of 10 s. The supernatant was obtained by centrifuging the homogenate at 4000× *g* (30 min, 4 °C) and was utilized for enzyme activity assay. The supernatant also served as the blank solution in place of sodium citrate buffer. Enzyme activity was assessed in a mixture of 250 µL supernatant, 250 µL of 0.1% Brij 35, and 250 µL of a reaction buffer composed of 340 mM sodium acetate, 4 mM Na_2_EDTA, 60 mM acetic acid, and 8 mM DTT, at pH 5.5. The substrate solution (20 µM Z-Phe-Arg-AMC) was preheated under uniform conditions, and 250 µL of the substrate mix was added for 2 min at 40 °C. The reaction was conducted for 10 min at 40 °C and terminated by the addition of 1 mL of a termination buffer (100 mM sodium acetate, 100 mM acetic acid, 100 mM trichloroacetic acid, pH 4.3). The supernatant was analyzed for fluorescence intensity using a microplate reader, following centrifugation of the reaction mixture at 12,000× *g* (10 min, 4 °C), with excitation and emission wavelengths set at 370 nm and 460 nm, respectively. Cathepsin B and L activity was quantified in units (U) as the increase in fluorescence per milligram of protein per unit time at 40 °C.

#### 2.7.2. Total Collagen Content

Total collagen was determined in accordance with ISO [38] and following the methodology of Ismail, Hwang [36]. Approximately 4 ± 0.05 g of sample was dissolved in 30 mL of 3.5 M H_2_SO_4_ for 16 h at 105 °C, filtered, and made up to 500 mL using DIW. A total of 1 mL of diluted solution was transferred into a glass cylinder and filled with 99 mL DIW, and 2 mL of the dilution was transferred into a sample tube, followed by the addition of 1 mL oxidation solution (comprising 1.41 g chloramines-T reagent and 100 mL of pH 6.0 buffered solution). The mixture was allowed to stand at 21 °C for 20 min. The buffer solution was obtained by solubilizing 90 g of sodium acetate trihydrate, 15 g of sodium hydroxide, and 30 g of citric acid monohydrate in 290 mL of 1-propanol, followed by dilution to 1 L with distilled water. A total of 1 mL of color reagent (19 g 4-dimethylamino benzaldehyde dissolved in 65 mL of 2-propanol and 35 mL of perchloric acid) was added and stirred. Subsequently, sealed tubes were submerged in hot water (60 °C/15 min). The absorbance of the chilled solution was quantified at 560 nm using UV–Vis spectroscopy (Agilent 8453, Santa Clara, CA, USA). A standard calibration curve was established using hydroxyproline at concentrations of 0, 1.2, 2.4, 3.6, and 4.8 µg, utilizing a coefficient of 8.

#### 2.7.3. Myofibrillar Fragmentation Index Assay

The changes in the myofibrillar fragmentation index (MFI) were assessed with the method by Xiong, Zhang [39]. Approximately 2 ± 0.05 g of sample was homogenized in MFI buffer consisting of 100 mM KCl, 11.2 mM KH_2_PO_4_, 8.8 nM K_2_HPO^4^, 1 mM EGTA, 1 mM MgCl_2_, and NaN_3_. The homogenate underwent centrifugation at 2400× *g* for 5 min, after which the supernatant was disposed of, and the specimen was immersed in 1 mL of DIW. Subsequently, a 1:4 (*v*/*v*) buffer solution was added, followed by further centrifugation. Absorbance was measured at 540 nm using an Agilent 8453 UV–Vis spectrophotometer (Santa Clara, CA, USA).

### 2.8. Histology of Meat Samples

For histology, samples were cut to 0.3 × 0.3 × 0.3 cm with a stainless-steel surgical knife along the muscle fibers to obtain transverse sections. These sections were sliced to a thickness of 1 micron using a Microm HM525 NX Cryostat (Fisher Scientific, Republic of Korea), dried, and stained with Harris Hematoxylin solution for 12 min. After rinsing in DIW for 10 min, coverslips were applied using glycerol gelatin. Slides were kept at room temperature for 12 h to dry, and images were taken using an Olympus CKX41SF Inverted microscope (Olympus Corp, Hachioji-Shi, Tokyo, Japan).

### 2.9. Microbiological Analysis

Approximately 10 ± 0.05 g of each sample was collected under sterile conditions and blended for 3 min with a Stomacher Lab-Blender 400 (UK). Buffered peptone water (BPW, Oxoid Ltd., UK) at 1:9 (*w*/*v*) dilution was added to the sample. Furthermore, plates were prepared (n = 3) through successive decimal dilutions in BPW for bacterial count. Following a 24–48 h incubation at 35–37 °C, total viable counts (TVC) of mesophilic bacteria were evaluated using Oxoid CM463 standard plate count agar. Total coliform counts (TCC) were quantified using sterilized pipettes and MacConkey agar after 24 to 48 h incubation at 36 °C. Total yeast and mold counts (TYMC) were assessed by culturing aerobically on potato dextrose agar (Merck 1.10130 Millipore, Sigma Aldrich, Auckland, New Zeland) for 48 h at 30 °C.

### 2.10. Taste Characteristics Assay by Taste Sensing System

Instrumental sensory flavor and taste were evaluated using a TST (INSENT SA402B Electric Sensing System, Atsugi, Japan) in accordance with the procedure outlined by Ismail, Hwang [40]. The TST consisted of sensor arrays, electrodes, data processing software, and specified synthetic lipid membranes. All calculated values were examined after the stabilization of the membranes in a standard meat taste (SMT) solution. The SMT formulation had 0.01% lactic acid (sourness), 0.25% monosodium glutamate (umami), and 0.0005% quinine hydrochloride (bitterness). Approximately 50 ± 0.05 g of each sample was combined with 200 mL of boiling DIW (95 °C, 20 min), then centrifuged at 1000× *g* (15 min, 4 °C). The sediment was separated using cellulose filter paper, and the resultant solution was preserved at −70 °C for subsequent analysis using TST.

### 2.11. Statistical Analysis

All analyses were conducted in triplicate, except for TPA, where five replications were taken. The outcomes of the above analyses were documented as the mean value and standard error of the mean (SEM). A one-way analysis of variance (ANOVA) was conducted using SAS 9.4 (SAS Institute, Cary, NC, USA). For multiple comparisons, Duncan’s multiple range test was applied. A *p*-value ≤ 5% was considered significant.

## 3. Results

### 3.1. Characterization of Taylor Cone, Bead, and Nanofiber Fabrication

**Figure 2:**
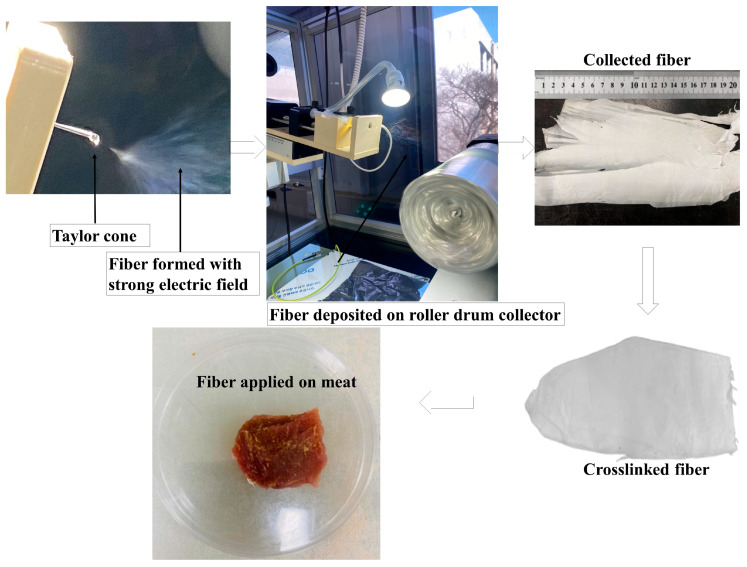
Taylor cone (conical shaped liquid droplet), fiber formation, fiber deposition, and application of collected fiber in beef.

Figure 2 and Supplementary Table S1 reveal the ES run results during NF fabrication and the resulting Taylor cone (TC), nanofiber (NF), and bead formation.

In the GE samples (30%), elevated flow rates (1.0 mL/h), faster rotation (1000 rpm), longer distance (18–24 cm), and larger needle size (22 G) consistently yielded bead formation without the presence of NF. Conversely, with a lower flow rate (0.5–0.6 mL/h), smaller needle (21 G), 500–600 rpm, and 14–16.5 cm, TC and NF were produced without beads. GTEP NF was produced at a reduced flow rate (0.6 mL/h) and the same distance as GE (15–15.5 cm), especially at a voltage of 22 kV. Enhanced concentrations of GTEP (2% and 3%) resulted in the formation of NF at a diminished distance (12 to 12.5 cm) and lower voltages (20–21 kV). An injector angle of 24° and a rotor speed of 500 rpm were ideal for NF production across all blend compositions. In compositions at elevated flow rates (0.8 to 1.0 mL/h), bead formation occurred, presumably due to inadequate stretching forces to mitigate surface tension effects [8].

### 3.2. Morphological Assessment

#### 3.2.1. HGGTNF Physical Structure

**Figure 3:**
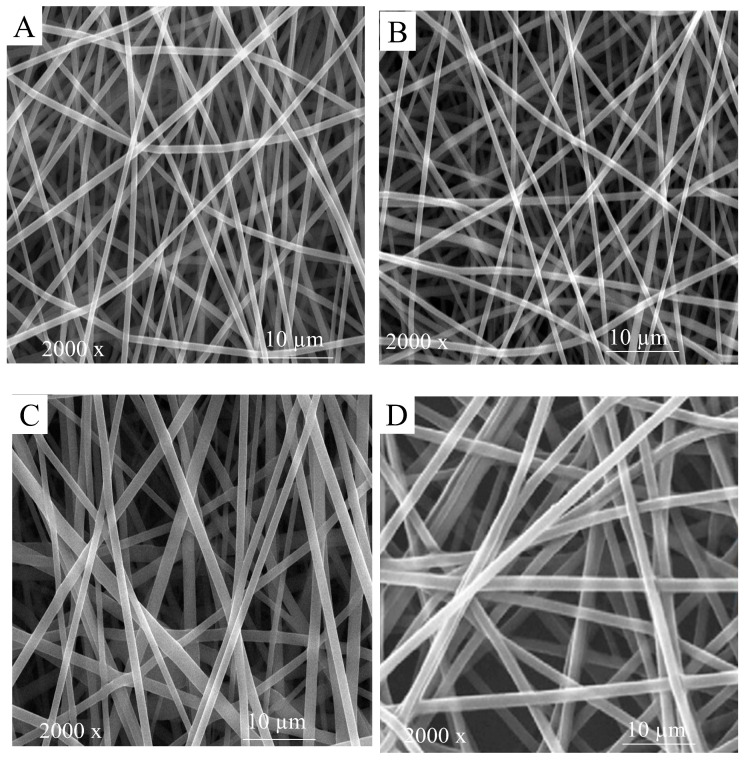
Electrospun hybrid GE/GTEP nanofiber (HGGTNF). (**A**) SEM image of gelatin outer layer nanofiber (NF); (**B**) SEM image of HGGTNF 1%; (**C**) SEM image of HGGTNF 2%, and (**D**) SEM image of HGGTNF 3%.

Uniform and smooth NFs improve mechanical qualities, enhancing strength and stretchability, along with an enhancement in the efficacy of NFs as a packaging material [8]. A homogenous structure facilitates the regulated release of GTEP, guaranteeing prolonged antibacterial and antioxidant activities [41]. Figure 3 shows the HGGTNF structures at 2000× magnification. The images indicate that the HGGTNFs were uniformly dispersed. Smooth NFs guarantee uniform distribution across the entire surface of the meat, optimizing the AP efficacy [42]. Notably, GE NF (3A), HGGTNF 1% (3B), HGGTNF 2% (3C), and HGGTNF 3% (3D) showed increasing fiber diameter with increasing levels of GTEP. These structural modifications influenced the mechanical properties of the fibers, including strength and elasticity, rendering them essential for comprehending fiber performance as AP [25].

#### 3.2.2. In Vitro Degradation

**Figure 4:**
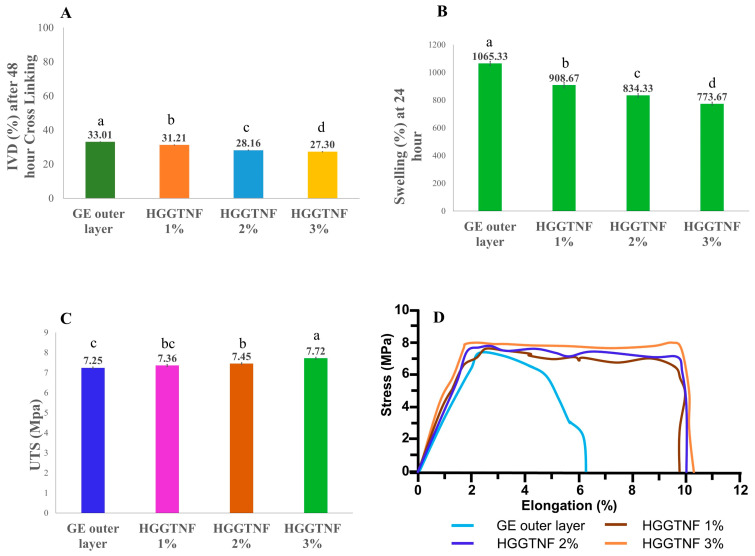
Stability of the HGGTNF. (**A**) In vitro degradation (IVD) status of gelatin outer layer nanofiber (NF) and HGGTNF (1–3%) at 48 h of crosslinking (CRL); (**B**) Swelling condition of GE outer layer NF and HGGTNF (1–3%) at 24 h; (**C**) Ultimate tensile strength of the HGGTNF (1–3%) in comparison to GE outer layer NF; and (**D**) Stress–strain curve for different samples. ^a–d^ Different superscript letters indicate significant differences (*p* < 0.05) within the samples.

The application of water-soluble polymers in the production of NF leads to their rapid breakdown upon exposure to biological fluids; thus, it must be crosslinked to enhance stability and [43] make it suitable for AP on meat surfaces. As shown in Figure 4A, all NF layers remained in a stable form with decreased degradability (around 28–33%) after 48 h of CRL. Previous studies indicated that gelatin-based NFs attain minimal stability after only 24 h of CL [44]; that is why, to attain better results, a longer CRL of 48 h was used in the present study.

#### 3.2.3. Swelling Parameters

The swelling test serves as a definitive assessment for evaluating the HGGTNF AP’s fluid absorption capacity, as illustrated in Figure 4B. It was observed that the outer layer nanofiber containing GE exhibited swelling of 1065.33 ± 21.62%, whereas HGGTNF 1%, 2%, and 3% showed 908.77 ± 13.30%, 843.67 ± 11.98%, and 773 ± 13.02% swelling, respectively. The addition of GTEP significantly reduced the swelling percentage with increasing concentration (*p* = 0.0001). Due to the hydrophilic nature of GE, the outer layer resulted in a higher swelling percentage [44]. Conversely, the addition of GTEP reduced swelling in HGGTNF. Previous studies suggest that the addition of nanoparticles in GE NFs usually reduces the swelling ratio [45].

#### 3.2.4. Tensile Strength

The tensile test is an indicator of the mechanical strength of NFs. As illustrated in Figure 4C, the outer layer GE NF had an ultimate tensile strength (UTS) of 7.25 ± 0.04 MPa, whereas, with increasing GTEP concentrations, the UTS of HGGTNF significantly increased (*p* = 0.0003), reaching 7.36 ± 0.05, 7.45 ± 0.04, 7.72 ± 0.05 at 1%, 2%, and 3% GTEP, respectively. The stress–strain curve of the samples is shown in Figure 4D. The increase in UTS with higher GTEP concentrations demonstrates the reinforcing influence of the nanoparticles [46]. Similar results were obtained with GE NF when ZnO nanoparticles were added to form HENF [44].

### 3.3. Nutritional Profile

The nutrient properties of the samples are outlined in Table 1. DM content exhibited considerable variations (*p* = 0.0001) between treatments, with the maximum in PEP (35.68 ± 0.03), followed by HGGTNF (34.04 ± 0.03, 34.49 ± 0.03, and 34.67 ± 0.02 at 1%, 2%, and 3% concentrations, respectively). Moisture content decreased progressively with increasing HGGTNF concentrations (65.92 ± 0.03, 65.51 ± 0.02, and 65.63 ± 0.02 at 1%, 2%, and 3%, respectively).

This tendency indicates that GTEP increased water retention characteristics. Furthermore, they may preserve protein integrity, which is reflected in the increasing CP values along with increased HGGTNF levels (22.17 ± 0.02, 22.41 ± 0.02, and 22.50 ± 0.02 at 1%, 2%, and 3%, respectively) compared to PEP (21.11 ± 0.02). CF content exhibited a notable reduction (*p* = 0.0001) in the PEP group (10.98 ± 0.02), whereas HGGTNF groups exhibited an upward trend, with the peak value for HGGTNF at 3% (11.34 ± 0.03). Ash content increased markedly with increasing HGGTNF levels, reaching a maximum of 1.15 ± 0.03 in the 2% and 3% HGGTNF groups, while the PEP group had a minimal ash content (1.04 ± 0.02).

### 3.4. Fatty Acid Profile

Table 1 delineates the FA composition and the principal groupings of saturated FA (SFA), monounsaturated FA (MUFA), polyunsaturated FA (PUFA), and atherogenic index (AI). SFA content increased markedly (*p* = 0.0001) in all treatment groups relative to PEP. The PEP group had the lowest SFA level (29.8 ± 0.01), followed by HGGTNF at 1%, 2%, and 3% concentrations. MUFA levels exhibited notable alterations (*p* = 0.0001), with HGGTNF-treated groups exhibiting a dose-dependent elevation, peaking at 3% (54.22 ± 0.01), signifying enhanced lipid unsaturation. PUFA levels were significantly lower in the PEP group relative to the HGGTNF group (*p* = 0.0013), while HGGTNF 2% and 3% demonstrated comparatively elevated PUFA content (3.79 ± 0.03 and 3.93 ± 0.01, respectively) in contrast to other groups. The lowest AI was noted in the HGGTNF groups, indicating enhanced benefits against cardiovascular disease (CVD).

### 3.5. Meat Color Parameters

**Figure 5:**
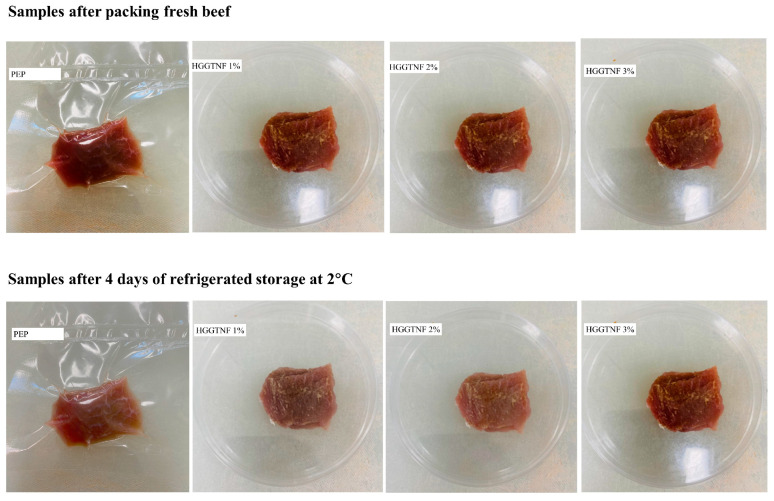
Physical appearance of beef sample with PEP and HGGTNF AP.

The physical appearance of the samples is illustrated in Figure 5 for clarity.

Table 2 presents the multiple colorimetric values across the different treatment groups. There were no significant changes in the Lightness (L)* values among the groups. HGGTNF treatments significantly (*p* = 0.0004) stabilized a* values, particularly at 1% (17.10 ± 0.21), 2% (18.14 ± 0.36), and 3% (19.41 ± 0.30), compared to the PEP group (16.55 ± 0.20), reflecting better redness preservation. An increased b* value was observed in the PEP group (8.88 ± 0.14), whereas HGGTNF samples exhibited stable b* values (8.55 ± 0.14, 8.53 ± 0.16, and 8.39 ± 0.14) in 1%, 2%, and 3% concentrations, respectively, hence averting the yellowish discoloration. These changes in a* and b* scores in the treated samples correspond with the observations of Yim, Jo [47], where the same increasing trend is observed with prolonged storage conditions in pork meat. Elevated Chroma (C*) and decreased hue angle (h°) indicate the maintenance of color vibrancy and inhibition of oxidative fading of food items [32]. This aligns with the present study results, where HGGTNF resulted in higher C* values (19.13 ± 0.16, 20.02 ± 0.20, and 21.14 ± 0.20 at 1%, 2%, and 3% concentrations, respectively) compared to PEP (18.77 ± 0.21), revealing vibrant and rich colors. The h° signifies a transition towards yellowish or brownish color and was higher in PEP (27.37 ± 0.17) than in the HGGTNF groups, which showed a stabilizing tendency with reduced values as GTEP concentration increased (26.57 ± 0.16, 25.14 ± 0.23, and 23.44 ± 0.13 at 1%, 2%, and 3%, respectively), thereby mitigating unwanted chromatic deviations in the samples.

### 3.6. Physicochemical Qualities

Table 2 highlights the parameters comprising pH, DL, CL, ERV, and WHC. The meat pH usually ranges from 5.5 to 5.8 and gradually increases with storage. Previous research established that meat pH exhibits a positive correlation with its WHC [48].

In the present study, pH values exhibited a stable and standard trend across treatments, with PEP demonstrating the lowest (*p* = 0.0100) pH (5.66 ± 0.02) in comparison to the HGGTNF groups (5.71 ± 0.02, 5.70 ± 0.02, and 5.78 ± 0.01 for 1%, 2%, and 3% groups, respectively), which were within acceptable ranges. A diminishing (*p* = 0.0005) trend was observed in PL for HGGTNF 1% (90.28 ± 0.04), HGGTNF 2% (91.39 ± 0.06), and HGGTNF 3% (91.52 ± 0.05) compared to PEP (90.09 ± 0.06). An elevated pH reduces PL by improving protein–water interactions, as demonstrated in HGGTNF 2% and 3% samples. The DL was minimal in HGGTNF 3% (1.15 ± 0.01), implying the capacity of HGGTNF as a protective covering for the meat. In contrast, PEP exhibited a higher DL (1.21 ± 0.01). CL followed the DL and PL trends, showing a decrease with increasing HGGTNF concentration, reaching a minimum at 3% concentration (18.13 ± 0.03) compared to the other groups. The decrease in CL in HGGTNF 3% suggests a positive relation with water retention and thermal stability in the samples. ERV was maximal in PEP (20.55 ± 0.07) and exhibited a discernible decline with escalating GTEP concentration, attaining its maximum at HGGTNF 3% (19.09 ± 0.04). There was a decrease (*p* = 0.0001) in ERV across the HGGTNF groups compared to PEP (20.55 ± 0.07). A notable decrease in ERV with HGGTNF indicates improved retention of extractable chemicals within the meat matrix.

### 3.7. Oxidative Degradation Parameters

The oxidative degradation insights are shown in Table 2. The PEP and HGGTNF 1% exhibit the highest FFA (0.14 ± 0.01, 0.14 ± 0.02) and POV (1.16 ± 0.07, 1.16 ± 0.06), respectively, signifying the accumulation of primary oxidation products. Higher concentrations of GTEP in HGGTNF 2% and 3% indicate improved oxidative stability. TBARS is a crucial indicator for secondary oxidation products such as malondialdehyde (MDA). TBARS values are markedly elevated (*p* = 0.0006) in PEP (0.29 ± 0.01), indicating increased lipid oxidation compared to the HGGTNF groups, where progressively decreased TBARS levels are observed (0.26 ± 0.01, 0.25 ± 0.01, and 0.23 ± 0.01) at 1%, 2%, and 3% concentrations, respectively.

### 3.8. Meat Textural Parameters

**Figure 6:**
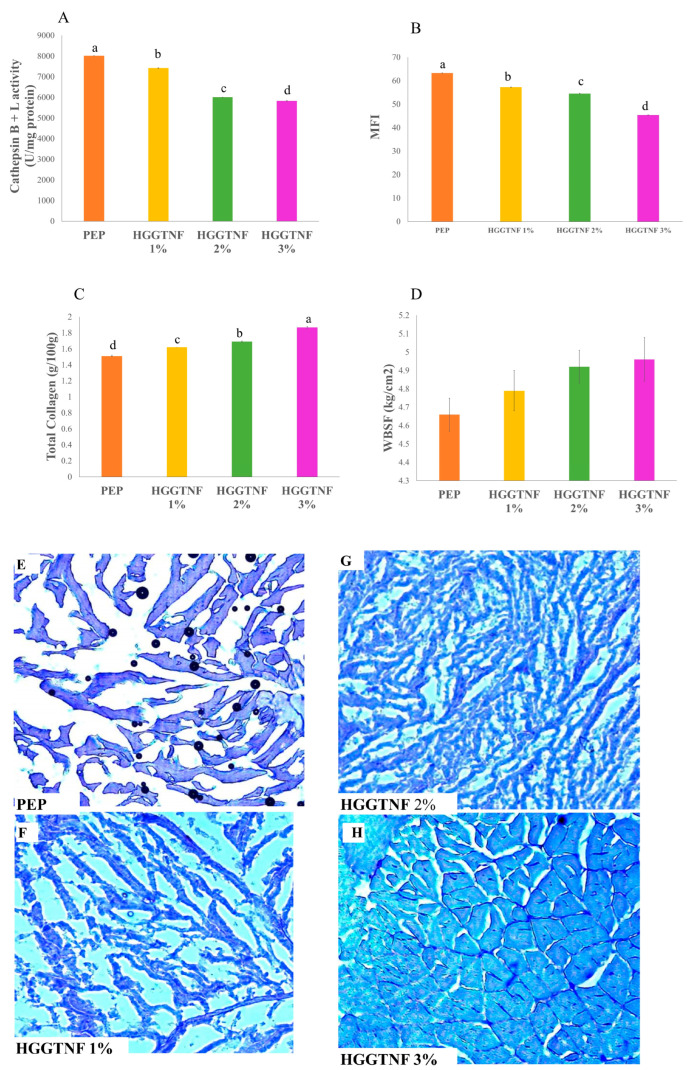
Muscle degradation studies of the samples. (**A**) Cathepsin B + L activity; (**B**) Hardness of the meat samples; (**C**) Total collagen content in meat samples; (**D**) Myofibrillar fragmentation index (MFI) of the samples; (**E**) Histology of samples packed with polyethylene; (**F**) Histology of samples packed with HGGTNF 1%; (**G**) Histology of samples packed with HGGTNF 2%; and (**H**) Histology of samples packed with HGGTNF 3%. ^a–d^ Different superscript letters indicate significant differences (*p* < 0.05) within the samples.

The textural parameters are documented in Table 2. The WBSF (kg/cm^2^) values (Figure 6D) showed an increased trend in the HGGTNF groups compared to PEP, although it was statistically insignificant. The parameters studied in TPA are hardness (N), defined as the maximum force required to compress meat following the initial compression, and springiness(mm), measured as the capacity of the sample to revert to its original form. Cohesiveness refers to the extent to which the sample can be deformed prior to disintegration. The TPA trend followed the WBSF results, showing an increased (*p* = 0.0001) hardness (N) in the HGGTNF groups, indicating improved structural integrity of muscle tissues. The springiness values among all the groups were stable, with no notable changes, suggesting that the treatments did not influence the elastic properties of the samples. Gumminess was markedly influenced by the GTEP treatments (*p* = 0.0085). The PEP group demonstrated the lowest gumminess value (23.25 ± 0.26) compared to the HGGTNF groups, where the 3% treatment had the highest value (27.35 ± 1.05). There were no significant changes in chewiness among the samples. Cohesiveness was markedly higher (*p* = 0.035) in the HGGTNF groups (0.65 ± 0.01, 0.70 ± 0.03, and 0.70 ± 0.03 at 1%, 2%, and 3%, respectively) than in PEP (0.62 ± 0.01).

### 3.9. Muscle Degradation Analysis

The enzymatic activity of Cathepsin B + L (Figure 6A) was maximal (*p* = 0.0001) in PEP (8009 ± 2.50), followed by HGGTNF treatments (7417 ± 16.06, 6009 ± 3.90, and 5822 ± 20.1 at 1%, 2%, and 3% concentrations, respectively). In our study, markedly elevated MFI values were observed in PEP (63.25 ± 0.07) compared to the HGGTNF groups (57.28 ± 0.07, 54.48 ± 0.17, and 45.42 ± 0.06 at 1%, 2%, and 3% concentrations, respectively, as shown in Figure 6B. Furthermore, there was a notable decrease (*p* = 0.0001) in total collagen (Figure 6C) in PEP (1.51 ± 0.01) relative to the HGGTNF 1%, 2%, and 3% groups (1.62 ± 0.01, 1.69 ± 0.01, and 1.87 ± 0.02, respectively); thus, HGGTNF reduced degradation in the present study. The reduced enzymatic activity resulted in higher WBSF in the treated samples (Figure 6D).

### 3.10. Histology of Meat Samples

The changes in muscle structures during preservation are shown in Figure 6E–H. This section integrates with the muscle degradation parameters discussed in Section 3.9. The degradation of MFP during preservation is well-established to occur through proteolytic enzyme activity, which consequently results in the enlargement of muscle cells due to the retention of moisture within the cells, resulting in cellular expansion [49,50]. The images from HGGTNF 2% (Figure 6G) and 3% (Figure 6H) in the present study exhibited fewer gaps between the muscle fibers and more fragmented muscle structure in the PEP (Figure 6E) and HGGTNF 1% (Figure 6F) samples, respectively. This image correlates with the MFI, where PEP and HGGTNF show the highest muscle degradation. Alternatively, HGGTNF 3% and 2% showed lower muscle degradation, respectively, which was reflected in the increased hardness in these samples.

### 3.11. Antimicrobial Effectiveness of Hybrid Nanofiber Mats

**Figure 7:**
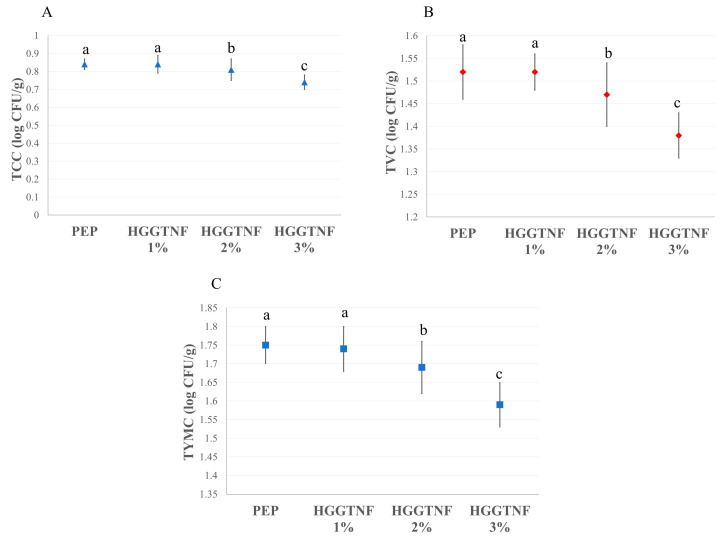
Microbiological quality of the meat samples packed with PEP and HGGTNF at different concentrations. (**A**) Total coliform count (TCC); (**B**) Total volatile count (TVC); and (**C**) Total yeast and mold count of the samples. ^a–c^ Different superscript letters indicate significant differences (*p* < 0.05) within the samples.

Figure 7 illustrates the efficacy of different doses of HGGTNF (1%, 2%, and 3%) in suppressing microbial proliferation over the experimental period in contrast to PEP. Figure 7A–C delineates the TCC, TVC, and TYMC counts, respectively, on day 4. The TCC in PEP (0.84 ± 0.03 log CFU/g) significantly (*p* = 0.0001) rose compared to HGGTNF 3%, which had the lowest count of 0.74 ± 0.04 CFU/g.

The lowest TVC count was observed in HGGTNF 3% (1.38 ± 0.05 log CFU/g) compared to PEP (1.52 ± 0.06 log CFU/g). A similar decreasing trend was observed in TYMC count for HGGTNF 3% (1.59 ± 0.06 log CFU/g) compared to PEP (1.75 ± 0.05 log CFU/g).

### 3.12. Taste Characteristics

**Figure 8:**
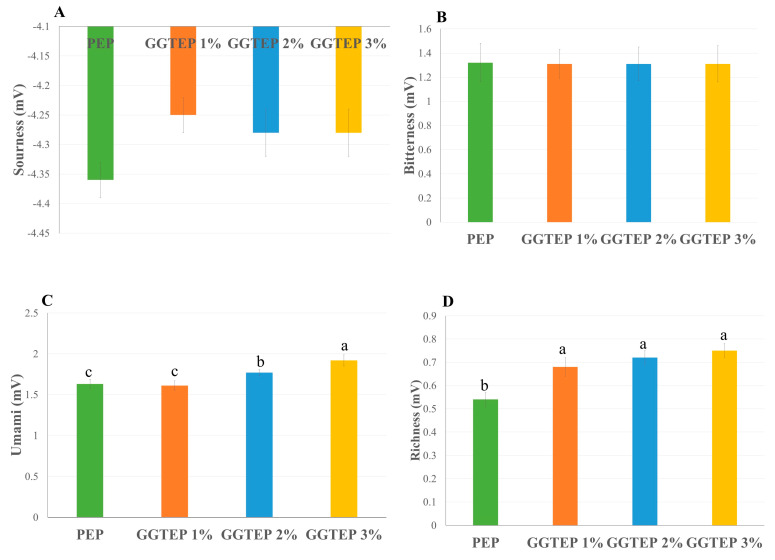
Taste traits of the samples were analyzed using an electronic taste testing system. (**A**) Sourness trait; (**B**) Bitterness trait; (**C**) Umami trait; and (**D**) Richness. PEP and HGGTNF (1–3%) samples. ^a–c^ Different superscript letters indicate significant differences (*p* < 0.05) within the samples.

The taste characteristics among the treatments are demonstrated in Figure 8. The sourness and bitterness traits showed no remarkable variations among the treatments. PEP exhibited the lowest umami trait (Figure 8C) (1.63 ± 0.06), whereas increasing (*p* = 0.0006) levels of HGGTNF enhanced the umami trait, with the highest value observed in the 3% group (1.92 ± 0.07). Richness (Figure 8D) also exhibited a notable increase (*p* = 0.0079) in HGGTNF 3% (0.75 ± 0.03) compared to PEP (0.54 ± 0.0).

## 4. Discussion

The majority of AP materials employed in the meat industry to extend shelf life include natural bioactive compounds with antioxidant and antimicrobial potency, such as eugenol, curcumin, tea polyphenols, etc. [18]. In the present study, GE-based HENF was fabricated using the ES technique with the incorporation of 1%, 2%, and 3% GTEP. GE was chosen in this study due to its superior gelation, thickening, encapsulation capacity for active ingredients, along with stabilizing characteristics that enhance the flavor, texture, and visual appearance of meat used in AP applications [16]. During this study, the ES technique was adopted as it can be fabricated at ambient temperatures (21–25 °C), preserving the active ingredients present in the biomaterials used as functional packaging [51]. Because of their advantages, ES HENF has emerged as promising, environmentally friendly, and sustainable alternative to traditional packaging materials [14].

HENF produced using ES usually possesses strong mechanical properties, such as porous structure, large surface area, and fine dimensions, which facilitate the use of active materials like GTEP in developing AP materials [52]. In the morphological assessment of the fabricated ES HENF in the present study, we observed an increase in fiber diameter with increasing levels of GTEP (1–3%). This can be explained through previous studies on GE/GTE NFs, where the addition of 0.1%, 0.5%, and 0.9% GTE resulted in increased fiber diameter [53]. Shao, Niu [54] observed that incorporating tea polyphenols at 0.5–1.5% under an applied voltage of 19–21 kV increased fiber diameter. In the present study, all NF combinations with GTE resulted in bead-free smooth fibers at 18–22 kV, which was similar to previous studies. The same study also concluded that the incorporation of higher concentrations of tea polyphenols increases solution viscosity and reduces solution conductivity, resulting in higher fiber diameter. The incorporation of GTEP also led to higher UTS with increasing concentration, which is due to the aforementioned factors. Previous studies have shown that HENF prepared with tea polyphenols exhibits higher tensile strength and significantly improved physical properties [55]. The same study also revealed lower degradation and swelling characteristics in HENF due to the incorporation of tea polyphenols. These results were similar to our present study, demonstrating the physical integrity of HGGTNF at higher concentrations. In the present study, improving the comprehension of the interactions between GE, GTEP, acetic acid, and CA through FTIR analysis could not be done and remains a limitation of this study. This has been recognized as a potential avenue for future research.

In the present study, HGGTNF preserved the nutritional profile during refrigerated storage, serving as an efficient AP material in comparison to PEP. Increased HGGTNF levels preserved protein integrity, as reflected in increased CP levels. Several recent studies have indicated that HENF is capable of protecting the nutritional and quality parameters of meat as a barrier material during storage [42,56,57]. Furthermore, the addition of antioxidants can protect proteins and fats from oxidation, improving the nutritional parameters during meat storage [35]. The enhancement in CF may be attributed to the antioxidative activities of HGGTNF, which can stabilize fat within the matrix, as observed in previous studies due to the potency of tea polyphenols [58]. The elevated ash in the HGGTNF groups indicates a binding contribution to the mineral composition of the samples. The decline in SFA and AI, along with the increase in MUFA, corroborates the evidence indicating the protective effect of unsaturated FA against CVD in the HGGTNF groups [59]. Furthermore, the notable stability in oleic acid and linoleic acid identified in the HGGTNF groups aligns with their established advantages in enhancing lipid profiles and diminishing LDL cholesterol levels. Tsuzuki [60] showed that the incorporation of antioxidants into fats and oils protected unsaturated FA (USFA). Rehim, Zahran [61] revealed that adding natural plant extracts to HGGTNF protected the FA profile in cheese during storage, which supports the efficiency of GTEP as a natural functional material.

The physicochemical properties in the present study demonstrate that the HGGTNF AP application was effective in preserving color parameters during refrigerated storage. The discoloration of meat during storage correlates with the oxidation process, elucidating the decline in a* and b* values throughout oxidation, with more significant alterations observed in the b* value [62]. Oxidative degradation during meat storage causes alterations in myoglobin, leading to a decrease in redness [63]. This indicates a darker sample due to oxidation of the preserved meat or food products. The stability in lightness values in HGGTNF-treated samples signifies its antioxidative effectiveness in preservation by reducing oxidative browning compared to the PEP sample. Furthermore, stabilizing a* values with HGGTNF indicates its ability to safeguard color-sensitive molecules from oxidative degradation. The incorporation of herbs and plant-derived antioxidants efficiently protects the color qualities of meat-based products [64]. Previous research indicated that the application of GTE-incorporated HENF affected different color parameters in beef cuts during refrigerated storage compared to the control [65]. In a similar study, Lorenzo, Sineiro [66] revealed that using tea extract in pork patties mitigated discoloration during refrigerated storage by diminishing the loss of redness and the augmentation of yellowness. They also concluded that tea extract could be a promising natural ingredient for substituting commercial antioxidants to improve the quality and composition of meat and meat products. Borzi, Torrieri [67] used GTE in polyamide film, which resulted in stable redness in beef at 23 days of storage compared to PEP. Sadakuzzaman, Rahman [35] conducted a study on adding synthetic antioxidants to beef and revealed a decrease in redness (a*) and an increase in yellowness (b*) in the control group compared to the antioxidant-treated groups during refrigerated storage, which is comparable to the findings of the current study. A previous investigation by Passos, Barreto [68] found that GTE-treated patties had a *h°* value similar to the control patty and suggested that GTE mitigated discoloration in the patties. This coincides with the present study, where GTEP preserved the vibrant color with increased Chroma and reduced *h*° at increased concentrations compared to PEP.

Maintenance of a stable pH with increasing concentrations of HGGTNF aligns with the research conducted by Kırmızıkaya, Karakaya [69], where GTE and other tea extracts were applied to minced beef, and the pH values of the treated samples remained within an acceptable range with increasing refrigerated storage period (5.44–5.86), which aligns with our present study. Another study on chicken patties with added GTE showed an increased but acceptable pH (5.81–5.82) compared to untreated samples [68]. The study by Montaño-Sánchez, Torres-Martínez [70] reported a pH of around 5.6–5.7 when pork meat was treated with chitosan and green tea water, which coincides with our present findings. Yoon, Bae [71] observed a similar pH pattern in meat treated with GTEP, consistent with the present findings. Based on the findings of Fang, Lin [72] on pork lean meat, pH levels of the GE-based AP increased during storage compared to unpacked control, suggesting superior preservation efficacy through the bacteriostatic and antioxidant properties of the films. The comparatively lower pH in the PEP sample was due to the breakdown of proteinaceous materials and the decomposition of amino acids [73,74]. Moreover, reduction in pH is also intricately linked to hydrolytic rancidity and the resultant formation of organic acids [75]. This implies that increasing levels of GTEP in HGGTNF AP provided an efficient barrier against decomposition and oxidation in beef. The pH findings of the present study also align with those documented by Lin, Mei [76]. In a study by Hamann, Puton [77], the application of GE GTEP film on sausage demonstrated a similar trend of stable pH by protecting the decomposition during storage.

Water retention is a critical commercial and sensory characteristic for meat quality [77], as meat with high water retention is often superior in preserving freshness, texture, and juiciness during consumption [78]. In the present study, water retention properties were better in the HGGTNF groups. Passos, Barreto [68] revealed a similar increase in water retention ability with increased incorporation of GTE in chicken patties. The lower DL in HGGTNF 3% indicates enhanced water retention characteristics, consistent with the previous results observed by Warner, Wheeler [79]. Bellucci, Bis-Souza [80] showed analogous outcomes in different meat products using natural plant extracts. In a recent study on plant-based meat alternatives by Han, Keum [81], the ERV was reduced with the addition of increasing levels of GTE. Several previous studies also support our findings. For instance, Yoon, Bae [71] showed that GTEP had no adverse effect on the physicochemical parameters of cured pork sausage. In another five-day storage study conducted by Gao, Hsu [55], GTE-based HENF was applied to chicken meat, and the GTE-treated group showed better water retention compared to the untreated group. The untreated group had a marked deformation from moisture loss, followed by a dull appearance compared to the GTE group. The overall freshness was compromised in the control group due to water loss. From the above discussions, it can be concluded that GTEP incorporation in GE-based HENF can be efficiently applied as an AP material to preserve the water retention qualities of beef and to preserve the integrity of appearance during refrigerated storage.

Lipid oxidation products, including hydroperoxides and their breakdown metabolites, contribute to the yellowing of meat, and peroxide concentrations signify the initial stages of lipid oxidation [82]. Moreover, lipid oxidation is a primary factor contributing to the decline in sensory attributes and shelf life of meat products [83]. In the present study, reduced FFA and POV values in HGGTNF at increasing concentrations indicate its ability to inhibit the onset of lipid oxidation by neutralizing free radicals [84]. Moreover, GTE efficiently blocks enzymes that facilitate fat oxidation, thereby minimizing excessive fat oxidation [85]. GTE is rich in polyphenols, flavonoids, and catechins [86], which are potent antioxidant substances and could be the main reason for the reduction in oxidation in the HGGTNF groups [87]. Yoon, Bae [71] found that elevated GTEP concentrations reduced lipid oxidation, suggesting a replacement of synthetic antioxidants like nitrite and ascorbate. The lower MDA values in the HGGTNF groups with increasing concentration suggest successful suppression of lipid peroxidation, probably attributed to the phenolic and flavonoid constituents in GTEP. This is supported by the findings of Passos, Barreto [68], who reported that 0.5% GTE in chicken patties had an increased TBARS, while 1% GTE reduced TBARS after 7 days of refrigerated storage. Integrating GTE into potato starch films significantly reduced the oxidation of packaged fresh beef and prevented metmyoglobin production [88]. In a study on pork conducted by Zhou, Liu [89], following five days of refrigerated storage, unpacked samples had an MDA value of 0.57 mg MDA/kg, while GTE NF-treated samples showed significantly lower values compared to the control, which was attributed to the oxidation of unsaturated fatty acids. Siripatrawan and Noipha [90] reported that the combination of GTE with chitosan films elevated the antioxidant characteristics, hence enhancing the shelf life of pork sausages. Chaijan, Panpipat [91] indicated that a coating derived from whey protein isolate enriched with GTE significantly inhibited the formation of TBARS in steaks during refrigerated storage, thereby prolonging storage stability from 1 to 2 weeks. GTE was effective in reducing the TBARS content in minced beef during 7-day refrigerated storage compared to the untreated group [69]. In a separate study, Alav, Kutlu [25] investigated PVA/GTE NF as AP for kiwi fruit, where treated samples reduced MDA levels during cold storage compared to fresh samples. All of the above observations support the present study in favor of HGGTNF, which can be used in HENF AP to extend the shelf life of beef.

HENF treatment affected the WBSF, hardness, and gumminess after 4 days of storage. Although the WBSF value was insignificantly higher in the HGGTNF group, this trend was consistent with the hardness value, where a significant increase (*p* = 0.0001) was observed compared to the PEP group. The increased hardness in the HGGTNF groups suggests that the structure of the meat retained firmness due to the protective function of HENF as a scaffold [92]. Moreover, this can be explained by the preservation of structural integrity in muscle tissues, which aligns with the findings of Schulte, Johnson [93], who illustrated the influence of enzymatic alterations affecting meat hardness. Increased gumminess in the HGGTNF group signifies protection against proteolytic degradation and collagen breakdown, as emphasized in prior research [94]. Similar findings were observed by Han, Keum [81], who revealed increased hardness in plant-based patties with increased levels of GTE. However, the direct inclusion of GTE in meat products did not find any significant changes in textural profiles [68]. In the present study, the increased gumminess of HGGTNF-treated samples indicates that GTE treatment enhanced textural qualities, suggesting a positive interaction between HENF and the meat protein network [95]. These results demonstrate the protective activity of the HGGTNF group against muscle degradation, thereby improving textural quality. Furthermore, they indicate that the original TPA profile of beef can be preserved with the application of HGGTNF AP.

During the process of meat preservation, meat muscle tissue is susceptible to softening due to autolysis, which results from enzymatic degradation caused by bacterial activity [42]. Cathepsin B + L is regarded as the primary endogenous enzyme responsible for the breakdown of meat proteins during postmortem conditions [96,97]. Increased cathepsin activity corresponds directly with enhanced proteolysis, facilitating meat tenderization [98], as represented in the texture values in Figure 6D. This trend is usual, and according to Han, Keum [81], in a study on beef, cathepsin activity increased during a 14-day storage experiment. In the present study, HGGTNF assisted in stabilizing the compactness of the muscle matrix at higher GTEP concentrations than the PEP group. Cathepsins are cysteine proteases that efficiently degrade myofibrillar proteins (MFP) in acidic environments, particularly myosin heavy chains and actin [99]. An increased MFI in meat implies enhanced proteolysis of MFP [100]. In this study, muscle degradation parameters were preserved due to the addition of HGGTNF AP in the meat samples. Moreover, collagen degradation is crucial in diminishing meat hardness since it alleviates connective tissue [79]. These values support the HGGTNF’s effectiveness as an AP material.

In general, GE NF does not possess antibacterial capacity, but HENF, after incorporation of GTE, can exhibit significant antibacterial activities. In the present study, the decrease in TCC, TVC, and TYMC in HGGTNF samples compared to PEP is ascribed to the antibacterial characteristics of HGGTNF, which presumably disrupt the integrity of the bacterial cell wall and metabolic functions [35,101]. Prior research indicates that natural ingredients like essential oils demonstrate superior antibacterial efficacy [102,103]. Various plant extracts have medicinal potency, and films formulated using extracts of *Vernicia fordii* and *Phyllanthus urinaria* have shown significant antibacterial efficacy against prevalent foodborne pathogens [104]. Duan, Sun [105] examined GE/Chitosan/Curcumin ES NF against *S. aureus* and *E coli* and it demonstrated remarkable bactericidal efficacy. GTE possesses antibacterial properties by inhibiting microbe adhesion and destroying their cellular structures [106]. Moreover, GTE has long been known to inhibit the growth and multiplication of gram-negative bacteria, gram-positive bacteria, and fungi [107]. Siripatrawan and Noipha [90] observed that the integration of GTE into chitosan films improved antibacterial characteristics and prolonged the shelf life of pork sausages. In another study, GTE incorporation in chicken patties controlled microbial spoilage during refrigerated storage [68]. Moreover, previous research has demonstrated that GTE has potent antibacterial properties against coliforms and gram-positive *cocci* [108], a trend that was observed in the present study. Vodnar [109] experimented with 4% GTE in ham steaks and observed a reduction in *Listeria monocytogenes* from an initial count of 0.55 log CFU/cm^2^ at room temperature and 1.655 log CFU/cm^2^ after refrigeration. The antimicrobial efficacy of HGGTNF corresponds with earlier research showing that bioactive chemicals significantly diminished pathogen populations via membrane disruption [110]. This explains the strong antimicrobial ability of GTE in meat and meat products.

Enhancing the umami flavor and taste could be a unique value addition to meat and meat products for strengthening consumer acceptance and has been studied in recent studies on beef and beef products using TST [30,111,112]. GTE possesses elevated levels of catechin and has a strong umami flavor. Moreover, cysteine in GTE is a principal component accountable for sweet and umami intensity [113]. Taking into consideration the commercial aspect and consumer preference for beef, we have incorporated GTE into the HENF to explore the possibility of enhancing umami and richness traits. In the present study, meat samples treated with HGGTNF showed better umami and richness values in comparison to the PEP group. Han, Keum [81] observed that increasing GTE concentration markedly attained superior results compared to untreated groups with respect to the taste and flavor of meat products, with improved umami intensity. Tannin present in GTEP can extend the shelf life and tenderness of meat. Nevertheless, excessive quantities may add a bitter flavor [114]. In the present study, there was no adverse effect on bitterness among the samples. In another study by Schilling, Pham [64] on pork sausage, the addition of GTE resulted in higher consumer acceptance and savory/umami intensity compared to control and rosemary extract-treated groups. Increasing levels of GTE infused into chicken patties achieved higher sensory scores for flavor and texture [68], signifying the favorable characteristics of GTE as a flavor enhancer in meat and meat products. Better umami and richness values observed in previous studies using GTE align with the present findings.

The present study focused on exploring the incorporation of GTEP in GE-based HENF to enhance the shelf life of beef, and the above results support this hypothesis. GTE has previously been incorporated into different kinds of biomaterials like GE, PVP, PVA, cellulose, and chitosan to fabricate antibacterial and antioxidant APs for food materials, fruits, chicken, pork, and fish to prolong shelf life [25,68,70,71,85,90,102,115,116,117], but fewer studies have focused on beef quality traits. In the present study, it was revealed that GTEP has potential as an AP in combination with GE as HENF to maintain beef storage quality and taste traits. In the future, different kinds of tea polyphenols can be explored for NF, HENF, and edible film formation for AP packaging for all types of meat products.

## 5. Conclusions

In this study, GE with increasing levels of GTEP (1%, 2%, and 3%) was used to fabricate HENF using the ES technique. This AP was applied to Hanwoo beef to assess physicochemical, fatty acid profile, textural, microbiological, and taste traits during 4-day refrigerated storage at 2 °C. The physical and mechanical properties of HGGTNF AP were acceptable with increasing GTEP concentration, with 3% and 2% showing lower in vitro degradability and higher tensile strength, respectively. The results showed that HGGTNF (3% to 2%) possesses significant potential as AP for preserving the qualitative traits of beef in comparison to traditional PEP. Application of HGGTNF 3% and HGGTNF 2% demonstrated stable pH, better water retention, and superior oxidative stability and microbiological qualities compared to PEP and HGGTNF 1%. HGGTNF 2% and HGGTNF 3% notably demonstrated efficacy in lowering POV, TBARS, cathepsin, and MFI, thus protecting the integrity of the meat texture. In addition, HGGTNF 3% and 2% showed antibacterial efficacy with decreased levels of TCC, TVC, and TYMC more efficiently than other groups, suggesting their potential as an AP to enhance the shelf life of beef. Furthermore, there was a notable change in the umami and richness of the sample with HGGTNF 3%, which are important taste traits for consumers. These biodegradable HGGTNF have the potential to be utilized as AP for beef or other meats to preserve quality and taste traits during storage. Further investigation is warranted to evaluate the application of ES NF as AP on plant-based meat (PBM) [118] and hybrid cultured meat (HCM) [119] as emerging meat alternatives. HENF should be further explored as an efficient and environmentally sustainable substitute for conventional packaging.

## Figures and Tables

**Table 1:** Nutritional profile and fatty acid content of the samples.

Nutritional Parameters	PEP	HGGTNF 1%	HGGTNF 2%	HGGTNF 3%	*p* Value
DM	35.68 ± 0.02 ^a^	34.04 ± 0.02 ^c^	34.49 ± 0.03 ^b^	34.67 ± 0.04 ^b^	0.0001
Moisture	64.32 ± 0.02 ^d^	65.92 ± 0.02 ^a^	65.51 ± 0.02 ^c^	65.63 ± 0.03 ^b^	0.0001
CP	21.11 ± 0.03 ^d^	22.17 ± 0.02 ^c^	22.41 ± 0.02 ^b^	22.50 ± 0.02 ^a^	0.0001
CF	10.98 ± 0.02 ^d^	11.11 ± 0.02 ^c^	11.20 ± 0.02 ^b^	11.34 ± 0.03 ^a^	0.0001
Ash	1.04 ± 0.02 ^b^	1.08 ± 0.02 ^ab^	1.15 ± 0.02 ^a^	1.15 ± 0.03 ^a^	0.0206
**Fatty acid profile**	**PEP**	**HGGTNF 1%**	**HGGTNF 2%**	**HGGTNF 3%**	***p* Value**
Lauric acid (C12:0)	0.06 ± 0.01	0.06 ± 0.01	0.06 ± 0.01	0.06 ± 0.01	NS
Myristic acid (C14:0)	2.36 ± 0.00 ^d^	2.39 ± 0.01 ^c^	2.41 ± 0.01 ^b^	2.52 ± 0.01 ^a^	0.0001
Myristoleic acid (C14:1)	0.74 ± 0.01 ^b^	0.75 ± 0.01 ^b^	0.75 ± 0.01 ^b^	0.78 ± 0.01 ^a^	0.0181
Palmitic acid (C16:0)	19.46 ± 0.01 ^d^	19.81 ± 0.00 ^c^	19.98 ± 0.01 ^b^	20.24 ± 0.00 ^a^	0.0001
Palmitoleic acid (C16:1)	4.45 ± 0.00 ^d^	4.61 ± 0.01 ^c^	4.65 ± 0.01 ^b^	4.81 ± 0.00 ^a^	0.0001
Stearic acid (C18:0)	7.78 ± 0.01 ^d^	7.89 ± 0.01 ^c^	7.97 ± 0.01 ^b^	8.19 ± 0.01 ^a^	0.0001
Oleic acid (C18:1n9c)	46.17 ± 0.01 ^d^	47.94 ± 0.00 ^c^	48.11 ± 0.00 ^b^	48.64 ± 0.00 ^a^	0.0001
Linoleic acid (C18:2n6c)	2.79 ± 0.01 ^d^	2.89 ± 0.01 ^c^	2.95 ± 0.01 ^b^	3.07 ± 0.00 ^a^	0.0001
α-Linolenic acid (C18:3n3)	0.16 ± 0.01	0.16 ± 0.01	0.16 ± 0.00	0.16 ± 0.01	NS
Arachidic acid (C20:0)	0.16 ± 0.01 ^c^	0.16 ± 0.00 ^bc^	0.17 ± 0.00 ^b^	0.19 ± 0.01 ^a^	0.0138
arachidonic acid (C20:4n6)	0.30 ± 0.01 ^c^	0.31 ± 0.01 ^bc^	0.33 ± 0.01 ^ab^	0.34 ± 0.00 ^a^	0.0292
eicosapentaenoic acid (C20:5n3)	0.31 ± 0.02	0.32 ± 0.01	0.34 ± 0.03	0.35 ± 0.00	NS
C22:6n3	0.02 ± 0.00	0.02 ± 0.00	0.02 ± 0.00	0.02 ± 0.00	NS
SFA	29.8 ± 0.01 ^d^	30.29 ± 0.02 ^c^	30.57 ± 0.02 ^b^	31.18 ± 0.00 ^a^	0.0001
MUFA	51.35 ± 0.02 ^d^	53.29 ± 0.01 ^c^	53.50 ± 0.02 ^b^	54.22 ± 0.01 ^a^	0.0001
PUFA	3.57 ± 0.04 ^d^	3.69 ± 0.00 ^c^	3.79 ± 0.03 ^b^	3.93 ± 0.01 ^a^	0.0013
AI	0.40 ± 0.00 ^a^	0.39 ± 0.00 ^c^	0.39 ± 0.00 ^b^	0.39 ± 0.00 ^b^	0.0002

PEP, poly ethylene packed; HGGTNF 1%, hybrid gelatin nanofiber loaded with 1% green tea extract powder; HGGTNF 2%, hybrid gelatin nanofiber loaded with 2% green tea extract powder; HGGTNF 3%, hybrid gelatin nanofiber loaded with 3% green tea extract powder; SFA, saturated fatty acids; MUFA, monounsaturated fatty acids; PUFA, polyunsaturated fatty acids; AI, atherogenic index. ^a–d^ Different superscript letters within the same row indicate significant differences between treatments (*p* < 0.05). NS, non significant.

**Table 2:** Colorimetric, physicochemical, oxidative degradation, and textural characteristics of the samples.

Color Parameters	PEP	HGGTNF 1%	HGGTNF 2%	HGGTNF 3%	*p*-Value
L*	36.53 ± 0.07	36.58 ± 0.06	36.76 ± 0.19	36.78 ± 0.15	NS
a*	16.55 ± 0.2 ^c^	17.10 ± 0.21 ^c^	18.14 ± 0.36 ^b^	19.41 ± 0.30 ^a^	0.0004
b*	8.88 ± 0.14	8.55 ± 0.16	8.53 ± 0.16	8.39 ± 0.14	NS
C*	18.78 ± 0.24 ^c^	19.12 ± 0.21 ^c^	20.05 ± 0.30 ^b^	21.14 ± 0.30 ^a^	0.001
*h* (°)	28.2 ± 0.08 ^a^	26.58 ± 0.51 ^b^	25.21 ± 0.74 ^b^	23.39 ± 0.34 ^c^	0.0006
**Physicochemical attributes**					
pH	5.66 ± 0.02 ^b^	5.71 ± 0.02 ^b^	5.7 ± 0.02 ^b^	5.78 ± 0.01 ^a^	0.0100
DL (%)	1.21 ± 0.01 ^a^	1.20 ± 0.01 ^a^	1.17 ± 0.01 ^b^	1.15 ± 0.00 ^b^	0.0005
CL (%)	20.26 ± 0.03 ^a^	19.91 ± 0.01 ^b^	19.05 ± 0.04 ^c^	18.13 ± 0.03 ^d^	0.0001
ERV (mL)	20.55 ± 0.07 ^b^	20.52 ± 0.03 ^a^	19.27 ± 0.02 ^b^	19.09 ± 0.04 ^c^	0.0001
PL (%)	91.52 ± 0.05 ^a^	91.39 ± 0.06 ^a^	90.28 ± 0.04 ^b^	90.09 ^d^ ± 0.06 ^c^	0.0001
**Oxidative degradation parameters**					
FFA (%)	0.14 ± 0.01 ^a^	0.14 ± 0.02 ^a^	0.13 ± 0.01 ^ab^	0.12 ± 01 ^b^	0.0226
POV (meq/kg)	1.16 ± 0.07 ^a^	1.16 ± 0.06 ^ab^	1.15 ± 0.05 ^ab^	1.14 ± 0.06 ^b^	0.0407
TBARS (mg-MDA/kg)	0.29 ± 0.01 ^a^	0.26 ± 0.01 ^b^	0.25 ± 0.01 ^b^	0.23 ± 0.01 ^c^	0.0006
**Textural parameters**					
WBSF (kg/cm^2^)	4.66 ± 0.09	4.79 ± 0.11	4.92 ± 0.09	4.96 ± 0.12	NS
Hardness (N)	37.74 ± 0.21 ^b^	37.84 ± 0.14 ^b^	38.53 ± 0.14 ^a^	38.97 ± 0.14 ^a^	0.0001
Springiness (cm)	0.92 ± 0.01	0.93 ± 0.02	0.90 ± 0.02	0.87 ± 0.03	NS
Guminess (N)	23.25 ± 0.26 ^c^	24.60 ± 0.82 ^bc^	27.04 ± 1.0 ^ab^	27.35 ± 1.05 ^a^	0.0085
Chewiness (N/cm)	21.47 ± 0.20	22.95 ± 0.73	24.47 ± 1.17	23.85 ± 1.55	NS
Cohesiveness	0.62 ± 0.01 ^b^	0.65 ± 0.02 ^ab^	0.70 ± 0.03 ^a^	0.70 ± 0.03 ^a^	0.035
**Muscle degradation parameters**					
Cathepsin B + L activity (U/mg protein)	8009.80 ± 2.50 ^a^	7417.80 ± 16.06 ^b^	6009.80 ± 3.90 ^c^	5822.80 ± 20.16 ^d^	0.0001
Total Collagen (g/100 g)	1.51 ± 0.01 ^d^	1.62 ± 0.00 ^c^	1.69 ± 0.01 ^b^	1.87 ± 0.01 ^a^	0.0001
MFI	63.25 ± 0.07 ^a^	57.28 ± 0.07 ^b^	54.48 ± 0.17 ^c^	45.42 ± 0.06 ^d^	0.0001

PEP, poly ethylene packed; HGGTNF 1%, hybrid gelatin nanofiber loaded with 1% green tea extract powder; HGGTNF 2%, hybrid gelatin nanofiber loaded with 2% green tea extract powder; HGGTNF 3%, hybrid gelatin nanofiber loaded with 3% green tea extract powder; DL, drip loss; CL, cooking loss; ERV, extract release volume; PL, purge loss; POV, peroxide value; TBARS, thiobarbituric acid reactive substance; WBSF, Warner–Bratzler shear force. ^a–d^ Different superscript letters within the same row indicate significant differences between treatments (*p* < 0.05). NS, non significant.

## Data Availability

The original contributions presented in the study are included in the article/Supplementary Material, further inquiries can be directed to the corresponding author.

## Supplementary Materials

The following supporting information can be downloaded at: https://www.mdpi.com/article/10.3390/foods14101734/s1, Table S1: Electrospinning parameters.
