# Supplementary material for: Meat Quality Traits Using Gelatin–Green Tea Extract Hybrid Electrospun Nanofiber Active Packaging
Source: Foods. 2025 May 13;14(10):1734. doi: 10.3390/foods14101734 (PMC12111436; doi:10.3390/foods14101734)
Supplement: Supplementary file 1 [file foods-14-01734-s001.zip › foods-3605663-supplementary.pdf]

Supplementary Table S1. Optimization of electrospinning parameters to fabricate bead-free nanofibers.

**NF Type**	***h*** **(%)**	***t*** **°C**	**GE/GTEP Conc (%)**	**LV (ml)**	**NS (G)**	**V (kV)**	**Angle (°)**	**d (cm)**	**R (rpm)**	**FL (mL/h)**	**Taylor Cone**	**Fiber**	**Bead**
GE Outer layer	80-85	20-25	30/0	10	18	22.5	35	24	1000	1	X	X	√
GE Outer layer	80-85	20-25	30/0	10	21	21	32	22	900	0.9	X	X	√
GE Outer layer	80-85	20-25	30/0	10	21	18.9	26	18	800	0.8	√	√	X
GE Outer layer	80-85	20-25	30/0	10	21	18.2	24	16	600	0.7	√	√	X
GE Outer layer	80-85	20-25	30/0	10	21	18	24	14.5	500	0.6	√	√	X
GGTEP 1%	80-85	20-25	30/1	10	23	22	28	16	1000	1	X	X	√
GGTEP 1%	80-85	20-25	30/1	10	23	21.5	26	15	800	0.8	X	X	√
GGTEP 1%	80-85	20-25	30/1	10	21	21.5	26	15	800	0.7	X	X	√
GGTEP 1%	80-85	20-25	30/1	10	21	22.2	26	15.5	500	0.6	√	√	X
GGTEP 1%	80-85	20-25	30/1	10	21	22	24	15	600	0.6	√	√	X
GGTEP 2%	80-85	20-25	30/2	10	23	24	28	18	1000	1	X	X	√
GGTEP 2%	80-85	20-25	30/2	10	23	23	28	16	800	0.6	X	X	√
GGTEP 2%	80-85	20-25	30/2	10	21	22.5	26	14	600	0.6	√	√	X
GGTEP 2%	80-85	20-25	30/2	10	21	21.5	25	12.5	500	0.6	√	√	X
GGTEP 2%	80-85	20-25	30/2	10	21	21	25	12	600	0.7	X	X	√
GGTEP 3%	80-85	20-25	30/3	10	25	18.5	24	16	600	0.7	X	X	√
GGTEP 3%	80-85	20-25	30/3	10	23	18	24	15	600	0.7	X	X	√
GGTEP 3%	80-85	20-25	30/3	10	21	19	25	14	500	0.7	√	√	√
GGTEP 3%	80-85	20-25	30/3	10	21	20	26	12.5	500	0.7	√	√	X
GGTEP 3%	80-85	20-25	30/3	10	21	21	26	12	600	0.6	√	√	X

GE, Gelatin, GGTEP, Gelatin and Green tea extract powder, H= Humidity, T= Temperature, LV= Syringe load volume, NS= Nozzle size, V=Voltage, R= Drum rotation, d= Distance from needle to rotating drum collector, R= Drum rotation speed, FL= Flow rate of the solution
